# Phosphate Uptake and Its Relation to Arsenic Toxicity in Lactobacilli

**DOI:** 10.3390/ijms25095017

**Published:** 2024-05-04

**Authors:** Daniela Corrales, Cristina Alcántara, María Jesús Clemente, Dinoraz Vélez, Vicenta Devesa, Vicente Monedero, Manuel Zúñiga

**Affiliations:** 1 Lactic Acid Bacteria and Probiotics Laboratory, Instituto de Agroquímica y Tecnología de Alimentos (IATA-CSIC), Av. Agustín Escardino 7, 46980 Paterna, Spain; dcorrales@iata.csic.es (D.C.); crisalba@iata.csic.es (C.A.); 2 Next-Generation Approaches for Integrative Food Toxicology Group, Instituto de Agroquímica y Tecnología de Alimentos (IATA-CSIC), Av. Catedràtic Agustín Escardino 7, 46980 Paterna, Spain; maria.jesus.clemente@uv.es (M.J.C.); deni@iata.csic.es (D.V.); vdevesa@iata.csic.es (V.D.)

**Keywords:** *Lactiplantibacillus plantarum*, *Lacticaseibacillus paracasei*, arsenate, arsenite, phosphate transporters, two-component system, PhoP

## Abstract

The use of probiotic lactobacilli has been proposed as a strategy to mitigate damage associated with exposure to toxic metals. Their protective effect against cationic metal ions, such as those of mercury or lead, is believed to stem from their chelating and accumulating potential. However, their retention of anionic toxic metalloids, such as inorganic arsenic, is generally low. Through the construction of mutants in phosphate transporter genes (*pst*) in *Lactiplantibacillus plantarum* and *Lacticaseibacillus paracasei* strains, coupled with arsenate [As(V)] uptake and toxicity assays, we determined that the incorporation of As(V), which structurally resembles phosphate, is likely facilitated by phosphate transporters. Surprisingly, inactivation in *Lc. paracasei* of PhoP, the transcriptional regulator of the two-component system PhoPR, a signal transducer involved in phosphate sensing, led to an increased resistance to arsenite [As(III)]. In comparison to the wild type, the *phoP* strain exhibited no differences in the ability to retain As(III), and there were no observed changes in the oxidation of As(III) to the less toxic As(V). These results reinforce the idea that specific transport, and not unspecific cell retention, plays a role in As(V) biosorption by lactobacilli, while they reveal an unexpected phenotype for the lack of the pleiotropic regulator PhoP.

## 1. Introduction

Inorganic arsenic [iAs: arsenite, As(III) and arsenate, As(V)] represents a significant health threat. It is estimated that a population of more than 100 million people are exposed to elevated amounts of this metalloid throughout the world, mainly via drinking water [1]. Owing to the severe adverse effects of iAs, international agencies recommend reducing dietary exposure [2]. In situations where achieving this goal is not feasible, such as in populations with no access to uncontaminated water sources, employing alternative approaches may alleviate the effects of iAs exposure. Strategies focused on diminishing iAs entry into the systemic circulation or reducing its toxicity have the potential to minimize damage.

Intestinal damage caused by heavy metals can be responsible in some instances for other systemic pathologies or contribute to increased entry of the toxicant in the organism. Strains of lactic acid bacteria have been postulated as agents (probiotics) aimed to prevent this adverse effect [3,4,5]. Selected strains of lactobacilli have been shown to alleviate inflammation, oxidative stress, increased intestinal permeability, and the accumulation of metals upon oral exposure through food or drink in animal models [6,7]. However, assays in human populations are still very scarce [8,9]. The chelation or sequestration of cationic toxic metals by negatively charged molecules of the cellular envelope has been hypothesized as a mechanism responsible for the observed positive effects. Additionally, the activation of anti-inflammatory and antioxidant pathways of the host possibly also plays a role [10,11]. In the case of iAs, which is an anionic pollutant, protective effects of probiotics on animal models have also been observed [12,13,14]. Strains of lactobacilli have been reported to complex iAs and remove it from aqueous solutions with varying efficacies [15]. However, owing to its anionic nature, the chelation or surface binding of iAs is presumed to play a less important role compared to metallic cations. The low ability of some lactobacilli strains to take up or retain the anionic forms of inorganic metalloid As has been previously described [16]. Other processes, in addition to iAs sequestration, are probably at play.

**Figure 1:**
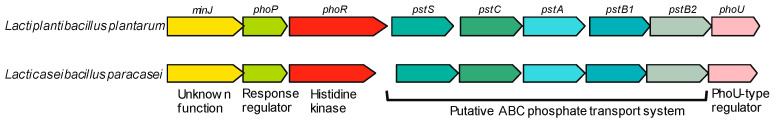
Schematic representation of *pho* and *pst* clusters harbored by *Lc. paracasei* and *Lp. plantarum*.

The sequestration or uptake of chemical contaminants by microorganisms can, in some cases, reside in microbial internalization mediated by specific transport systems [4]. Mechanisms to counteract the intrinsic toxicity of iAs usually rely on the uptake of As(V) molecules and reduction to As(III) by arsenate reductase enzymes (Ars). Subsequently, this As species, which is even more toxic than As(V), can be expelled from the cells by specific As(III)-detoxifying pumps [17]. Although these mechanisms are not common or widespread in lactobacilli, they have been genetically identified in some strains [18,19]. As(V), which structurally resembles phosphate, can possibly enter the cells by the same natural mechanisms as phosphate [20]. The most studied phosphate uptake system in bacteria is the widely distributed high-affinity–low-capacity phosphate transporter of the ABC-type family (*pstSCAB*) [21]. This system is composed by a surface-bound phosphate-binding protein (PstS), transmembrane transport subunits (PstCA), and cytoplasmic ATPase subunits (PstB), which energize transport. An additional auxiliary gene, *phoU*, encoding a small regulatory protein, is often present (see, for example, Figure 1). In many instances, the encoding genes are clustered with the genes encoding a cognate two-component system (TCS), PhoBR (named PhoPR in some model Gram-positive bacteria such as *Bacillus subtilis*), putatively involved in sensing the extracellular phosphate concentration and controlling the expression of phosphate-regulated genes. Phosphate uptake by the Pst ABC system is linked to the activity of PhoBR in the model organism *E. coli* (Supplementary Figure S1). Under low-phosphate conditions, the conformational changes derived via transport through PstCA and ATP hydrolysis by PstB are sensed by PhoU, which interacts with the sensor kinase PhoR, triggering its phosphorylation function on PhoB, the response regulator of the TCS [22]. PhoB controls the expression of phosphate-regulated genes, including the *pst* genes, activating their transcription under conditions of low phosphate availability [23].

A PhoPR TCS has been studied in *Lacticaseibacillus paracasei* BL23 (originally named TC04; PhoP, LCABL_10480, PhoR, LCABL_10490), showing that mutating the response regulator *phoP* resulted in slow growth and acid sensitivity in an MRS medium [24]. This TCS was adjacent to a *pstSCAB_1_B_2_-phoU* gene cluster (Figure 1), but this operon has not been studied in any member of the *Lactobacillaceae* family. The inspection of available genome sequences within the *Lactobacillaceae* family showed this typical organization (Supplementary Figure S2; [25]), in which a *phoU* gene also follows a *pstSCAB* cluster. This operon generally carried two tandem copies of the ATPase-encoding gene *pstB* (*pstSCAB_1_B_2_*), and *phoPR* genes were found upstream.

In this study, our aim was to elucidate the mechanisms responsible for the binding of As in lactobacilli and their possible contribution to As toxicity. To this end, the binding capacities of a set of lactobacilli strains were determined. From this set, strains of *Lc. paracasei* and *Lactiplantibacillus plantarum* were selected for subsequent analyses. Through the construction of mutants, we demonstrated that As(V) is likely taken up by the Pst ABC transport system in *Lp. plantarum*. These mutants were also used to determine if a correlation existed between As uptake and sensitivity. Additionally, we made an unprecedented finding, reporting a link between the activity of the PhoP response regulator and As(III) sensitivity in *Lc. paracasei*.

## 2. Results

### 2.1. As(V) and As(III) Incorporation by Lactobacilli Strains

A survey of a set of lactobacilli strains was carried out in order to estimate their capacity of retaining different As chemical species. The percentages of retention after the incubation of high-density cell suspensions in phosphate-buffered saline (PBS) with As(V) or As(III) or with the organic arsenic form dimethylarsinic acid (DMA) were below 10% in all cases (Table 1).

In order to gain insight into the mechanisms involved in As uptake, we focused on *Lc. paracasei* and *Lp. plantarum* species, since they are amenable to genetic manipulation. For As(V), we hypothesized that incorporation in lactobacilli could take place through specific phosphate transporter(s). Therefore, to assess the contribution of the Pst transporter to As(V) incorporation, we decided to construct mutants in the transmembrane phosphate permease PstC in *Lp. plantarum* Lpp+, *Lp. plantarum* WCFS1 [26], and *Lc. paracasei* BL23. In addition, insertional inactivations of *phoP* and *phoU* were also attempted. While *pstC*, *phoP*, and *phoU* mutants of the WCFS1 and BL23 strains were successfully obtained, for unknown reasons and despite several attempts, only *pstC* disruptants of the Lpp+ strain could be achieved.

### 2.2. Mutation of pst Genes Coding for ABC Phosphate Transporters Impacts As(V) Uptake and Toxicity

**Figure 2:**
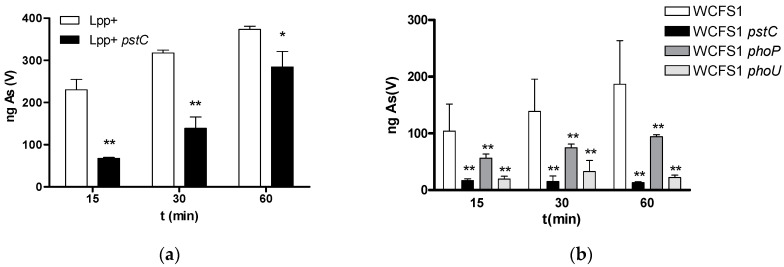
The incorporation of As(V) in *Lp. plantarum* strains and their derivative mutants affected in *pst* and *pho* genes (**a**) *L. plantarum* Lpp+; (**b**) *Lp. plantarum* WCFS1. Columns represent average amounts of As retained by 300 μL of bacterial cells at an OD_595_ of 10 when exposed to 5 mg/L of As(V) (*n* = 3). Error bars represent standard deviations. * (*p* < 0.05); ** (*p* < 0.01), statistical differences with respect to the wild-type strain for each time point.

We performed time-course As(V) incorporation assays in the *Lp. plantarum* strains defective in *pstC*, *phoP*, or *phoU* genes (Figure 2). In these experiments, the absence of phosphate allowed As(V) uptake by the cells, including the WCFS1 strain, which had showed low As(V) uptake when phosphate was present (Table 1). As(V) incorporation by the WCFS1 strain was lower compared to the Lpp+ strain. However, the mutation of the PstC component of the phosphate ABC transporter in Lpp+ resulted in reduced As(V) uptake, while it was almost abolished in the WCFS1 *pstC* derivative (Figure 2). The capacity of the WCFS1 *phoU* strains to incorporate As(V) was comparable to that of the *pstC* strain, whereas a *phoP* mutant had an intermediate behavior. The capacity to incorporate As(V) by *Lc. paracasei* BL23 under these conditions was very low and did not show differences between the wild-type or the *pstC*-defective strain.

**Figure 3:**
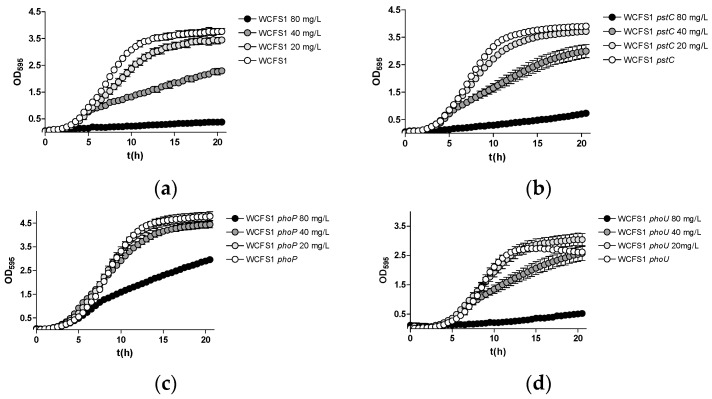
Growth curves of *Lp. plantarum* WCFS1 and different *pst*- and *pho*-derived mutants in the MEI medium (high phosphate) with different amounts of As(V) indicated in mg/L. (**a**) Wild type; (**b**) *pstC* mutant; (**c**) *phoP* mutant; (**d**) *phoU* mutant.

**Figure 4:**
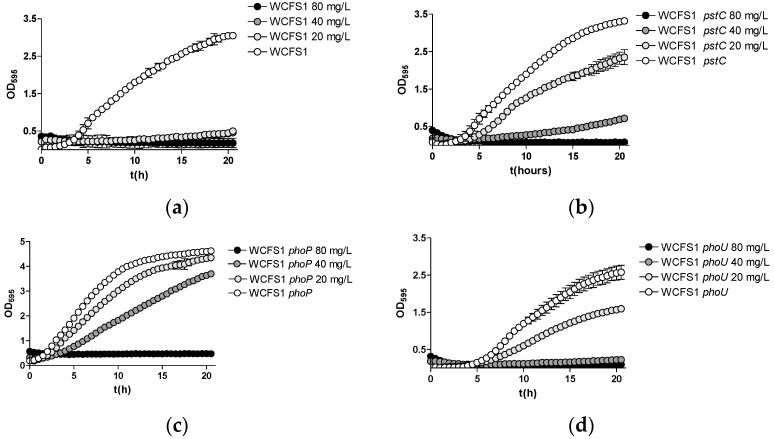
Growth curves of *Lp. plantarum* WCFS1 and different *pst*- and *pho*-derived mutants in the LP-MEI medium (low phosphate) with different amounts of As(V) indicated in mg/L. (**a**) Wild type; (**b**) *pstC* mutant; (**c**) *phoP* mutant; (**d**) *phoU* mutant.

We next assessed the toxicity of As(V) by culturing the different strains in growth media with a high or low phosphate concentration (MEI and LP-MEI medium, respectively) containing different concentrations of As(V). The toxicity and the effects of the mutations varied between both *Lp. plantarum* strains. However, it was observed that the WCFS1 strain exhibited lower As(V) toxicity in high-phosphate conditions (Figure 3) compared to low-phosphate conditions (Figure 4). Furthermore, when growth experiments were conducted under low-phosphate conditions in WCFS1, the inactivation of either *pstC* or *phoP* enhanced resistance to As(V). The inactivation of *phoU* in strain WCFS1 also led to a slight increase in resistance against As(V) (Figure 4). The effects of the mutations were much less evident when the strains were grown in the MEI medium. This supports the idea that the presence of phosphate had a protective effect on As(V) toxicity, likely through competition with a common incorporating system. Furthermore, a *pstC* mutation displayed no effects on As(V) toxicity in the *Lp. pantarum* Lpp+, irrespective of the phosphate concentration in the medium (Supplementary Figure S3). Similar to WCFS1, increased resistance was found in the BL23 *pstC* strain, only under low-phosphate conditions (Supplementary Figure S3).

### 2.3. Mutations in the phoPR TCS Result in Increased As(III) Resistance in Lc. paracasei

While conducting the As toxicity tests in the mutant lactobacilli strains, we made an unexpected observation. The disruption of *phoP* in the *Lc. paracasei* BL23 strain resulted in increased resistance to As(III). The disruption of *phoP* in BL23 had been obtained by plasmid integration, which can result in strong polar effects on the expression of downstream *phoR* or *pst* genes. In order to avoid this, we constructed an in-frame internal deletion of *phoP* in BL23, which resulted in the expression of a mutant PhoP protein devoid of 189 amino acids [PhoP(Δ13-201)], which included the phosphorylatable Asp-52 residue.

**Figure 5:**
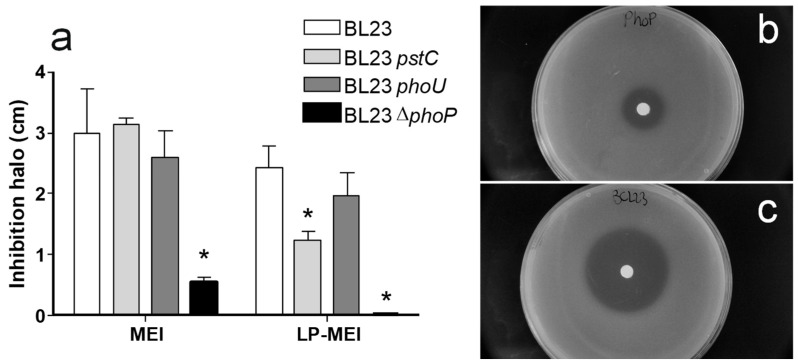
(**a**) Diameters of inhibition haloes of As(III) on MEI (high phosphate) and LP-MEI (low phosphate) plates seeded with different strains of *Lc. paracasei* BL23 wild-type and mutants in *pst* and *pho* genes. Columns represent average diameters (*n* = 3). Error bars represent standard deviations. Asterisks indicate statistical differences compared to the wild type for each growth condition (*p* < 0.01). (**b**) The inhibition halo of As(III) on the MEI plate seeded with the *Lc. paracasei* Δ*phoP* mutant. (**c**) *Lc. paracasei* BL23.

Figure 5 depicts the diameters of the inhibitory halos, resulting from spotting filter papers impregnated with As(III) on lawns of bacteria in a medium with high or low phosphate contents. While clear growth inhibition was observed for the wild type, no such inhibition haloes were evidenced for the Δ*phoP* strain under low-phosphate conditions, while they were strongly reduced under high-phosphate conditions. Compared to the wild type, this reduction in the diameter of haloes caused by As(III) could also be observed in the *pstC* and *phoU* strains only under low-phosphate conditions. However, the effect of these mutations was weaker compared to Δ*phoP*.

**Figure 6:**
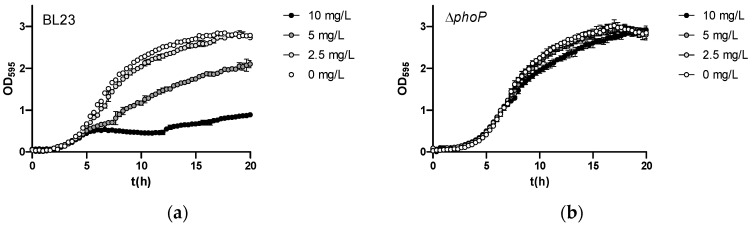

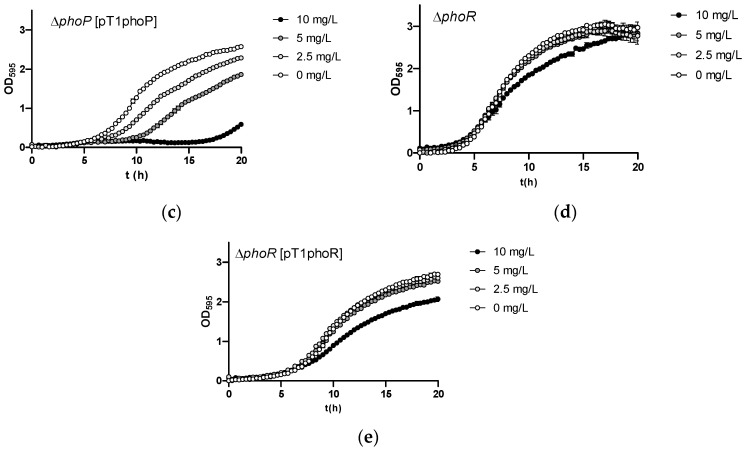
Growth curves of *Lc. paracasei* BL23 and *phoP* or *phoR* mutants and their complemented strains in the MEI medium with different amounts of As(III). (**a**) BL23 (wild type); (**b**) Δ*phoP*; (**c**) Δ*phoP* [pT1phoP], complemented strain; (**d**) Δ*phoR*; (**e**) Δ*phoR*[pT1phoR], complemented strain.

Growth assays confirmed the reduced sensitivity to As(III) of the Δ*phoP* strain (Figure 6). Furthermore, the complementation of the Δ*phoP* strain by expressing wild-type PhoP from a plasmid restored As(III) sensitivity. This excluded the possibility that polar effects on adjacent genes due to mutation (insertion or deletion) were responsible for the observed phenotype in BL23 *phoP* strains. The As(III) resistance phenotype upon *phoP* mutation was exclusive for *Lc. paracasei*, as in the equivalent *Lp. plantarum* mutant strains, no apparent changes in As(III) sensitivity were observed (Supplementary Figure S4).

Functionality of TCS usually requires the concerted action of a sensor histidine kinase and a response regulator [28]. Therefore, we next explored whether eliminating PhoR in the BL23 strain resulted in a similar As(III) resistance phenotype as that detected in *phoP* cells. To this end, a new strain where *phoR* was completely deleted by double recombination was obtained. The toxicity of As(III) was reduced compared to the wild-type strain, reaching levels similar to that of a Δ*phoP* strain. Although transforming this strain with a plasmid bearing *phoR* did not fully complement the inactivation of *phoR* (Figure 6), these results suggested that resistance to As(III) was possibly linked to genes under the control of PhoRP.

### 2.4. No Differences in As(III) Oxidation Are Observed in Lc. paracasei phoP or phoR Mutants

**Figure 7:**
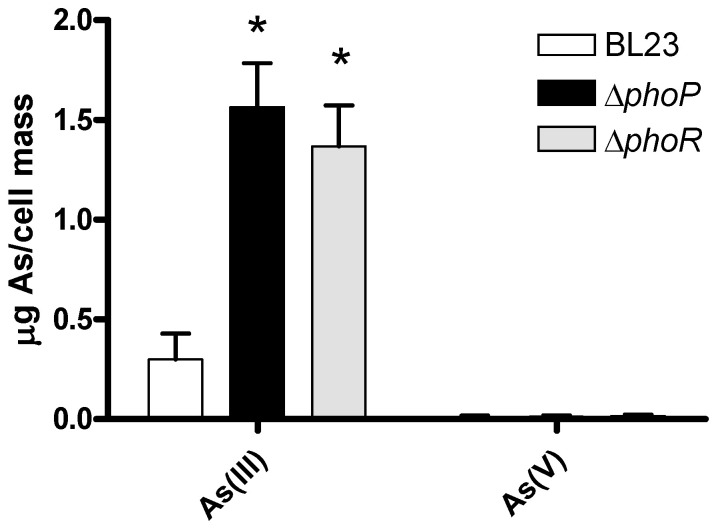
Inorganic As speciation in bacterial cell pellets of different *Lc. paracasei* strains. Cells were grown in the MEI medium containing 5 mg/L of As(III), and the contents of As(III) and As(V) in the bacteria were quantified and are referred to as μg of As per gram of wet mass (*n* = 3). Error bars are standard deviations. Asterisks indicate statistical differences compared to the wild type (*p* < 0.01).

The fact that eliminating PhoP triggered resistance to As(III) in *Lc. paracasei* was puzzling, as this inorganic arsenic species does not resemble phosphate and its links with phosphate transport/metabolism are not obvious. We hypothesized that being a pleiotropic transcriptional regulator, the lack of PhoP could lead to changes in the bacterial cell capacity to retain As(III), leading to decreased toxicity of the metalloid. When *Lc. paracasei* strains were incubated with 5 mg/L of As(III) at an OD_595_ of 10, they showed low capacity to bind As(III) and most of the As(III) added to the cell suspensions was recovered by an initial washing step in both the wild-type and Δ*phoP* strains (98.4 ± 0.5% and 96.4 ± 3.0%, respectively). In these experiments, As(III) retained by bacteria after two washing steps was somewhat higher for the Δ*phoP* strain (56.3 ± 40.2 ng of As per ml of cells at an OD_595_ of 10) compared to the wild type (4.9 ± 2.3 ng of As per ml of cells at an OD_595_ of 10). This excluded the possibility that reduced surface interaction or binding resulted in reduced toxicity in the *phoP* strains. A second possibility was that in *phoP* or *phoR* strains, a likely oxidation of As(III) to the less toxic As(V) species was enhanced or taking place, thus affecting the toxicity of iAs. We tested this possibility by performing experiments in which As(III) was present during growth in the MEI medium in the wild-type, *phoP*, and *phoR* strains of BL23 and determining the speciation of different iAs forms present in the cells. The oxidation of As(III) to As(V) did not take place in these assays, as the proportion of As(V) was always very low and similar for all strains (between 0.9 and 2% of total As; Figure 7). This indicated that increased As(III) resistance upon *phoP* or *phoR* deletion probably resides in mechanisms different to As(III) oxidation. In these experiments, the capacity of mutant bacteria to retain As(III) was higher compared to the wild type. However, it has to be considered that in these experiments, owing to the intrinsic As(III) toxicity, the wild-type strain displayed lower growth compared to Δ*phoP* and Δ*phoR* strains.

## 3. Discussion

Strains of lactobacilli have demonstrated the ability to incorporate inorganic arsenic (iAs), predominantly in the As(V) form [15,29]. They have been proposed as potential tools for protecting against metal toxicity, and even as a means of eliminating it from water or other beverages by using these food-grade microorganisms, akin to similar applications suggested for addressing other contaminating or toxic substances like mycotoxins [15,30]. Our results have shown a low capacity of the accumulation of the different As chemical species by lactobacilli. For As(V), this last circumstance could be attributed to the fact that phosphate structurally resembles this As species and may interfere with its incorporation [16]. Furthermore, previous studies reported the need of live cells for As(V) incorporation [16]. Therefore, the widespread presence of phosphate may limit the capacity for As(V) accumulation, hindering its effective utilization. This situation mirrors that observed with other toxic metals, such as mercury, which typically forms complexes with thiolated compounds in food matrices [31]. These compounds alter the affinity of probiotic strains for mercury, thereby interfering with the chelation process.

We have demonstrated that the As(V) uptake capability of *Lp. plantarum* WCFS1 may be associated with the Pst ABC-type phosphate transporter. The presence of *pstSCAB* clusters in lactobacilli (see Supplementary Figure S2) suggests that this transporter could be a major phosphate uptake system in this bacterial group, although no prior studies on it have been reported for these microorganisms. While Pst transporters have been extensively characterized in bacteria and thoroughly studied in model organisms such as *E. coli*, other phosphate transporters like the Pit permeases have also been identified for phosphate uptake [21,32]. The existence of alternative and additional phosphate transporter(s) might explain the varied effects of *pstC* inactivation on As(V) incorporation in the two strains of *Lp. plantarum* used. In *Lc. paracasei* BL23, a strain that does not display any remarkable As(V) incorporation capacity, mutating *pstC* also resulted in increased As(V) resistance when cells were cultured in a medium with low phosphate. The effects of *phoP* and *phoU* mutations in WCFS1 and BL23 strains do not have a straightforward interpretation and may indicate that, in the absence of these putative regulators, potential changes in PstSCAB expression may impact As(V) incorporation.

In *E. coli*, strains harboring a *phoU* mutation exhibit the deregulation of phosphate transport, allowing for the incorporation of higher amounts of phosphate [33]. In this bacterium, PhoU serves a regulatory function, linking the transport activity through Pst components to the auto-phosphorylation of the sensor kinase PhoR (Supplementary Figure S1). This mechanism provides a means of sensing external phosphate concentrations and subsequently regulating PhoP activity [21,23]. Presently, the exact role of PhoU in lactobacilli remains unknown. While PhoU is present in some bacteria possessing PstSCAB transporters and PhoPR orthologues, it is absent from others, including some lactobacilli. It is also remarkable that in some members of the *Lactobacillaceae* (e.g., species of *Lacticaseibacillus*, *Lactiplantibacillus*, *Latilactobacillus*, *Loigolactobacillus*, *Levilactobacillus*, *Lentilactobacillus*, *Liquorilactobacillus*, *Agrilactobacillus*, or *Secundilactobacillus*), a gene encoding a putative protein with eight transmembrane segments and a C-terminal cytoplasmic PDZ domain (LCABL_10470 in *Lc. paracasei* BL23) is always located upstream of *phoP*, forming a likely operon structure with *phoPR* (Supplementary Figure S2). Therefore, the possible function of this gene in the Pho-Pst regulatory network deserves further investigation.

In addition to the observed acid sensitivity and reduced growth rate in the *Lc. paracasei* BL23 *phoP* strain in the MRS medium [24], we have uncovered an unexpected As(III) resistance phenotype. Our investigation ruled out the possibility that the BL23 *phoP* strain was incorporating less As(III) or promoting its oxidation to As(V). In fact, *Lc. paracasei phoP* and *phoR* mutants accumulated more As(III) compared to the wild-type strain, although As(III) uptake in *Lc. paracasei* occurs at very low levels. The reasons for this effect are unknown. In *E. coli*, the *pho* regulon is well characterized. This regulon includes genes dedicated to phosphate uptake and phosphate scavenging under conditions of limited phosphate supply, such as genes encoding phosphatases and the *pst* genes [23]. However, owing to the metabolic relevance of phosphate, a defect in PhoP, and hence in cell phosphate supply, may have important pleiotropic effects. How a defect in *phoP* in the BL23 strain increases As(III) resistance is still not known. A defect in the sensor-kinase-encoding gene *phoR* in *Lc. casei* BL23 also resulted in a resistance phenotype compared to *phoP*. This suggests that the resistance in a *phoR* mutant may be attributed to the absence of PhoP activation through phosphorylation due to the lack of its cognate kinase. However, the possibility that the deletion of *phoR* resulted in an altered expression of *phoP* cannot be excluded, as we could not confirm complementation when we transformed the Δ*phoR* strain with a plasmid bearing *phoR*.

As(III) is taken up in *E. coli* by the glycerol channel GlpF [34], but the encoding gene has not been identified as a member of the Pho regulon. *Lc. paracasei* BL23 encodes two putative GlpF homologs (LCABL_07210 and LCABL_08620). Unfortunately, no functional information is available on these genes, and their possible involvement in As(III) uptake or tolerance remains to be determined. Some bacteria encode redox enzymes and specific iAs-detoxifying transporters as a defense system against iAs toxic effects [35]. However, no specific As(III) pump, nor arsenate-reductase-encoding genes, can be identified in the *Lc. paracasei* BL23 genome that could be eventually overexpressed in the *phoP* mutant. Links between As(III) and PhoPR have been described in other bacteria, revealing other metabolic connections between As and phosphate metabolism. In *Halomonas* sp., the TCS PhoBR (homolog to PhoPR) regulates the expression of the *aioBA* genes coding for As(III) oxidases, which promote As(III) to As(V) conversion depending on phosphate availability [36]. In *Agrobacterium tumefaciens*, an antimonite [Sb(III)]-detoxifying mechanism that promotes Sb(III) oxidation to antimonate [Sb(V)] has also been described, mediated by the Sb(III) oxidase AnoA, which shows cross-reactivity with As(III), and whose expression is also controlled by phosphate through PhoB (PhoP) [37]. However, mutations in *phoB* in these bacteria lead to a reduced expression of these oxidases, which decreases As(III) conversion and Sb(III) detoxification, and *aioBA* or *anoA* homologous genes are not present in the BL23 genome.

Our results indicate that the protective effect of some lactobacilli on their hosts against As damage is likely unrelated to their capacity for As accumulation. Therefore, the potential beneficial effects of lactobacilli on iAs toxicity possibly involve other mechanisms, such as anti-inflammatory and antioxidant properties unique to certain strains. As an example, recent experiments in animal models showed that strains like *Lc. paracasei* BL23, which does not possess As(III) [nor As(V)]-binding abilities, reduce most of the intestinal toxic effects of As(III) exposure through drinking water in a murine model [12]. The involvement of phosphate transport in As(V) uptake by *Lp. plantarum* has also been evidenced. Our results highlight the importance of the PstSCAB system in phosphate metabolism in lactobacilli and point to the existence of alternative phosphate transporters.

We have also demonstrated that *Lc. paracasei* lacking *phoP* or *phoR* does not exhibit apparent As(III) detoxification (oxidation) but displays enhanced resistance to it. The characterization of the *pho* regulon in *Lb. paracasei* and the determination of transcriptomic and proteomic changes resulting from *phoP* elimination will contribute to understanding the alterations caused by the absence of this regulator, which triggers As(III) resistance. In particular, elucidating the cross-talk mechanisms associated with phosphate sensing via PhoPR, which in other microorganisms involve carbon, nitrogen, iron, potassium, sodium, and sulfur metabolism, as well as resistance to general stresses [38], will enhance our comprehension of this regulatory network.

## 4. Materials and Methods

### 4.1. Bacterial Culture Conditions

The lactobacilli strains used in this study (Table 1 and Table 2) were routinely grown in a de Man, Rogosa, and Sharpe (MRS) medium (BD Difco™. Thermo Fisher Scientific, Alcobendas, Spain) at 30 or 37 °C, under static conditions. For iAs toxicity assays, the MEI medium was employed containing (*w*/*v*) a 0.5% yeast extract, 0.5% tryptone, 0.4% K_2_HPO_4_, 0.5% KH_2_PO_4_, 0.02% MgSO_4_·7H_2_O, 0.005% MnSO_4_, 0.05% cysteine, 0.5% glucose, and 1 mL of Tween 80 per liter. When low-phosphate conditions were employed, no K_2_HPO_4_ and KH_2_PO_4_ were added to the MEI medium (LP-MEI medium). *E. coli* DH10B [F^−^ *endA*1 *recA*1 *galE*15 *galK*16 *nupG rpsL* Δ*lacX*74 Φ80*lacZ*ΔM15 *araD*139 Δ(*ara*,*leu*)7697 *mcrA* Δ(*mrr*-*hsdRMS-mcrBC*) λ^−^] was employed for cloning purposes and it was grown in an LB medium at 37 °C under strong agitation (200 rpm). For *E. coli* clone selection, ampicillin was used at 100 μg/mL. Erythromycin at 5 μg/mL was used for the selection of recombinant clones in lactobacilli. Solid media were made by adding 1.8% (*w*/*v*) agar.

### 4.2. Construction of Strains Mutated in pst and pho Genes

The oligonucleotides used in this work are listed in Supplementary Table S1. Chromosomal DNA from *Lp. plantarum* and *Lc. paracasei* strains was isolated with the DNA Isolation Kit for Cells and Tissues (Roche® Life Science, Basel, Switzerland). Internal fragments ranging from 300 to 500 bp from *pstC*, *phoP*, and *phoU* were amplified by PCR from chromosomal DNA with NZYTaq II DNA polymerase (NZYtech, Lisbon, Portugal). The obtained fragments were purified with the GFX PCR DNA and Gel Band Purification Kit (Cytiva, Chicago, IL, USA), digested with appropriate restriction enzymes, and cloned into the integrative plasmid pRV300 [39] digested with the same enzymes. The ligation mixtures were transformed into *E. coli* DH10B and recombinant clones were selected in LB agar plates with ampicillin, 40 μg/mL of X-gal, and 0.1 mM IPTG. The resulting plasmids were purified from *E. coli* with the NucleoSpin Plasmid Kit (Macherey-Nagel GmbH & Co KG, Dueren, Germany) and used to transform *Lp. plantarum* WCFS1, *Lp. plantarum* Lpp+, and *Lc. paracasei* BL23 to obtain disruption mutants by single cross-over integration. Plasmid integration at the correct locus was checked by PCR with one oligonucleotide that hybridized in the targeted gene outside the cloned fragment and an oligonucleotide hybridizing in the pRV300 plasmid.

To obtain Δ*phoP* and Δ*phoR* derivatives from the BL23 strain, fragments of 1 kb upstream and downstream of the desired deletion were synthesized by PCR with Phusion High-Fidelity DNA Polymerase (Thermo Fisher Scientific, Alcobendas, Spain). These fragments overlapped by 20 bp and they were fused by a second PCR reaction using them as templates. The obtained 2 kb fragments were digested with appropriate restriction enzymes and cloned into pRV300. The integrative plasmids thus obtained were used to transform *Lc. paracasei* BL23. Strains with a first integration of the plasmid in the chromosome were isolated on MRS plates containing erythromycin. One transformant was selected from each construction and they were grown for approximately 200 generations in the absence of antibiotics. Isolates in which a second recombination event took place, leading to plasmid excision, were selected by their erythromycin sensibility by replica-plating. Among the erythromycin-sensible clones obtained from each construction, the presence of the desired deletion was checked by PCR and confirmed by sequencing.

*Lp. plantarum* and *Lc. paracasei* were transformed by electroporation with a Gene Pulser apparatus (Bio-Rad, Hercules, CA, USA). *Lp. plantarum* electrocompetent bacteria were prepared in polyethylene glycol (PEG) 1500 as described [40] with some modifications. The bacteria were cultured in 50 mL of MRS supplemented with 1% (*w*/*v*) glycine to an OD_595_ of 0.4–0.6. After washing with 1 volume of cold 1 mM MgCl_2_, the cells were washed with a half volume of cold 30% PEG 1500 and resuspended in 500 μL of 30% PEG 1500. Cells were electroporated in 0.2 cm cuvettes at 1.5 kV, 25 μF, and 400 Ω, with 0.5–2 μg of purified plasmids, and resuspended in 1 mL of MRS. After incubation at 30 °C for 2 h, the transformed bacteria were plated on MRS plates containing 5 μg/mL of erythromycin and incubated at 30 °C for 48 h. *Lc. paracasei* BL23 was transformed by electroporation as previously described [41] and the transformants were isolated on MRS plates with 5 μg/L of erythromycin incubated at 37 °C.

### 4.3. Strain Complementation

The *phoP* and *phoR* genes from *Lc. paracasei* BL23 were amplified by PCR with Phusion High-Fidelity DNA Polymerase (Thermo Fisher Scientific, Alcobendas, Spain) and appropriate oligonucleotides (Supplementary Table S1). The purified PCR fragments were ligated to a BglII/SpeI-digested pT1NX [42] plasmid with the GeneArt™ Gibson Assembly EX kit (Invitrogen, Thermo Fisher Scientific, Alcobendas, Spain), leading to *phoP* and *phoR* genes in which their expression was under the control of the lactococcal P1 constitutive promoter, respectively. The products of the Gibson reaction were used to transform *Lactococcus lactis* MG1363 electrocompetent cells [43], and transformants were selected on M17 (Oxoid, Thermo Fisher Scientific, Alcobendas, Spain) agar plates containing 0.5% (*w*/*v*) glucose (GM17) plus 5 μg/mL of erythromycin. Plates were incubated for 48 h at 30 °C. Colonies were checked by PCR and positive clones bearing inserts were grown in 5 mL of GM17 for plasmid isolation with the NucleoSpin Plasmid Kit (Macherey-Nagel GmbH & Co KG, Dueren, Germany) with modifications. Cells were incubated in an STE buffer (20% sucrose, 10 mM Tris-HCl [pH 8.0], 10 mM EDTA, 50 mM NaCl) supplemented with lysozymes (1 mg/mL) for 30 min at 37 °C before cell lysis. Subsequent steps were carried out as indicated by the manufacturer. pT1NX derivatives carrying *phoP* and *phoR* were sequenced and these expression plasmids were used to transform *Lc. paracasei* Δ*phoP* and Δ*phoR* mutant strains, respectively, by electroporation.

### 4.4. As Toxicity, Incorporation, and Speciation Assays

As(V) (stock solution of 1000 mg/L, As_2_O_5_) was purchased from Merck (Merck KGaA, Darmstadt, Germany). The As(III) solution (1000 mg/L) was prepared by dissolving 1.320 g of As_2_O_3_ (Riedel-de Haën, Seelze, Germany) in 25 mL of KOH 20% *w*/*v*. After neutralization with 20% H_2_SO_4_ *v*/*v*, this solution was made up to a final volume of 1 L with H_2_SO_4_ 1% *v*/*v*. The DMA(V) [dimethylarsinic acid; (CH_3_)_2_AsNaO_2_·3H_2_O (Honeywell Fluka™, Thermo Fisher Scientific, Alcobendas, Spain)] stock solution was prepared in water.

The lactobacilli strains were cultured in MEI or LP-MEI media with different As(III) or As(V) concentrations in 96-well plates (200 μL per well) at 30 °C (*Lp. pantarum*) or 37 °C (*Lc. paracasei*) in a Spectrostar Nano plate reader (BMG-Labtech, Ortenberg, Germany). OD readings at 595nm were recorded every 30 min. As(III) inhibition assays on plates were carried out by placing Whatman 3MM filter paper discs (5 mm diameter) impregnated with 3 μL of As(III), 1000 mg/L, onto 90 mm MRS agar plates with an overlay of 5 mL of MRS with 0.8% agar containing 10^6^ CFU of different *Lc. paracasei* strains. After incubation for 24 h, the diameter of inhibition haloes was measured.

As(V), As(III), and DMA retention capacity in different lactobacilli was tested in cells from overnight cultures (5 mL) resuspended in phosphate-buffered saline (PBS) to an OD at 595 nm of 10. Cell suspensions were supplemented with 1 mg/L of As(V), As(III), or DMA, respectively, and incubated for 1 h at 37 °C. Cells were centrifuged at 5000× *g* for 10 min and washed with 5 mL of PBS. The As retained in the bacterial pellets was determined as indicated below.

For time-course As(V) incorporation assays, strains were cultured overnight in 50 mL of MRS, pelleted by centrifugation (5000× *g*, 10 min), and washed with 1 vol of 0.9% NaCl. Washed bacteria were resuspended in 0.9% NaCl, and OD at 595nm was adjusted to 10. Aliquots of the cell suspensions (1 mL) were incubated at 30 °C for 5 min in a water bath before As(V) was added to a final concentration of 5 mg/L. Samples of 300 μL were withdrawn at different time intervals and quickly filtered under vacuum using 0.45 μm nitrocellulose filters (Millipore). The filters were washed twice with 5 mL of 0.9% NaCl and air dried before As quantification.

The As(III) retention capacity of *Lc. paracasei* strains was estimated by measuring the amount of As(III) retained by 1 mL of cells at an OD at 595nm of 10. Cell suspensions were incubated with 5 mg/L of As(III) for 1 h at 37 °C in 0.9% NaCl. After centrifugation at 10,000× *g* for 5 min, bacterial pellets were washed twice with 1 mL of 0.9% NaCl, and As(III) in washing supernatants and the bacterial pellet was determined.

For total As determination in samples (bacterial-washing supernatants, cell pellets, and filters), after a dry ashing step, As quantification was carried out by flow injection–hydride generation–atomic absorption spectrometry (FI-HG-AAS), following the procedure described by Clemente et al. [44].

To determine As(III) oxidation to As(V) by *Lc. paracasei* cells, 50 mL of the MEI medium containing 5 mg/L of As(III) was inoculated with *Lc. paracasei* BL23 and their derived Δ*phoP* and Δ*phoR* mutants at an initial OD at 595nm of 0.01. After 16 h of incubation at 37 °C, cells were washed at 4 °C with cold 0.9% NaCl (4000× *g*, 10 min) and the bacterial pellets were kept at −20 °C until the analysis. iAs was extracted from bacterial pellets with 5 mL of 0.28 M HNO_3_ at 95 °C for 1.5 h [45] and the As(III) and As(V) contents were determined by HPLC with an anion exchange column (Hamilton PRP X100, 150 × 4.1 mm, particle size: 5 μm; Hamilton Bonaduz AG, Bonaduz, Switzerland) coupled to a PerkinElmer Nex-Ion™300X ICP-MS (PerkinElmer, Waltham, MA, USA) as described [46].

### 4.5. Statistical Analysis

One-way ANOVA with Tukey’s multiple comparison test and Student’s *t* test were carried out with GraphPad Prism 5.00 (GraphPad Software, Boston, MA, USA). Differences were considered statistically significant at *p* < 0.05.

## Figures and Tables

**Table 1:** Percentages of retention of As(III), As(V), or DMA by different lactobacilli strains.

Strain Designation and Collection Code	% As(III) Retention	% As(V) Retention	% DMA Retention
BL7 *Levilactobacillus brevis* DSMZ ^a^ 1268	0.48 ± 0.08	0.06 ± 0.01	0.09 ± 0.02
BL10 *Lactobacillus acidophilus* ATCC ^b^ 9224	3.28 ± 1.40	0.80 ± 0.18	0.74 ± 0.13
BL17 *Lactobacillus acidophilus* ATCC ^b^ 4356	3.99 ± 0.95	0.41 ± 0.27	0.88 ± 0.27
BL23 *Lacticaseibacillus paracasei* CECT ^c^ 5275	0.08 ± 0.07	0.09 ± 0.03	0.03 ± 0.02
BL36 *Levilactobacillus brevis* ATCC ^b^ 14869	3.01 ± 0.19	2.92 ± 0.05	0.35 ± 0.01
BL73 *Lactobacillus acidophilus* CNRZ ^d^ 55	2.60 ± 0.32	0.77 ± 0.42	0.83 ± 0.35
BL75 *Lactobacillus acidophilus* CNRZ ^d^ 21	3.27 ± 0.06	0.49 ± 0.12	0.51 ± 0.35
BL166 *Lactiplantibacillus plantarum* WCFS1 ^e^	1.26 ± 0.12	0.01 ± 0.03	0.47 ± 0.05
BL221 *Lactobacillus crispatus* M247 ^f^	1.20 ± 0.10	0.36 ± 0.14	0.24 ± 0.08
BL278 *Lactobacillus crispatus* DSMZ ^a^ 20584	1.38 ± 0.04	0.27 ±0.01	0.37 ± 0.04
BL279 *Lactobacillus acidophilus* CECT ^c^ 4529	1.25 ± 0.12	0.20 ± 0.09	0.41 ± 0.10
BL280 *Lactobacillus acidophilus* CECT ^c^ 4179	2.41 ± 0.15	0.41 ± 0.13	0.76 ± 0.41
Lpp+ *Lactiplantibacillus plantarum* ^g^	2.55 ± 0.16	5.70 ± 0.16	1.12 ± 0.09

The strains were incubated at 37 °C for 1 h with different as species (1 mg/L) at an adjusted OD_595nm_ of 10 in PBS. Cells were washed with one volume of PBS, and as retained by the cells was quantified. DMA, dimethylarsinic acid. ^a^ Deutsche Sammlung von Mikroorganismen und Zellkulturen; ^b^ American Type Strain Culture Collection; ^c^ Colección Española de Cultivos Tipo (CECT); ^d^ Centre National de Recherches Zootechniques (CNRZ); ^e^ [26]; ^f^ [27]; ^g^ Collection of the Lactic Bacteria and Probiotics Laboratory [Instituto de Agroquímica y Tecnología de Alimentos (IATA)].

**Table 2:** *Lp. plantarum* and *Lc. paracasei* strains and their derived mutants used in this study.

Strain	Genotype	Reference
*Lp. plantarum* WCFS1	wild-type	[26]
*Lp. plantarum* DC421	WCFS1 *pstC*::pRV300; *ery*^R a^	This work
*Lp. plantarum* DC423	WCFS1 *phoP*::pRV300; *ery*^R^	This work
*Lp. plantarum* DC425	WCFS1 *phoU*::pRV300; *ery*^R^	This work
*Lp. plantarum* Lpp+	wild-type	Laboratory stock
*Lp. plantarum* DC424	Lpp+ *pstC*::pRV300; *ery*^R^	This work
*Lc. paracasei* BL23	wild-type	CECT 5275
*Lc. paracasei* DC399	BL23 *pstC*::pRV300; *ery*^R^	This work
*Lc. paracasei* TC04	BL23 *phoP*::pRV300; *ery*^R^	[24]
*Lc. paracasei* DC398	BL23 *phoU*::pRV300; *ery*^R^	This work
*Lc. paracasei* DC487	BL23 Δ*phoP*	This work
*Lc. paracasei* DC488	BL23 Δ*phoP* [pT1phoP]; *ery*^R^	This work
*Lc. paracasei* DC489	BL23 Δ*phoR*	This work
*Lc. paracasei* DC490	BL23 Δ*phoR* [pT1phoR]; *ery*^R^	This work

^a^ Erythromycin resistance.

## Data Availability

The raw data supporting the conclusions of this article will be made available by the authors on request.

## Supplementary Materials

The following supporting information can be downloaded at: https://www.mdpi.com/article/10.3390/ijms25095017/s1.
