# Supplementary material for: Phosphate Uptake and Its Relation to Arsenic Toxicity in Lactobacilli
Source: Int J Mol Sci. 2024 May 4;25(9):5017. doi: 10.3390/ijms25095017 (PMC11084836; doi:10.3390/ijms25095017)
Supplement: Supplementary file 1 [file ijms-25-05017-s001.zip › Supplementary Figure S1-S4.pdf]

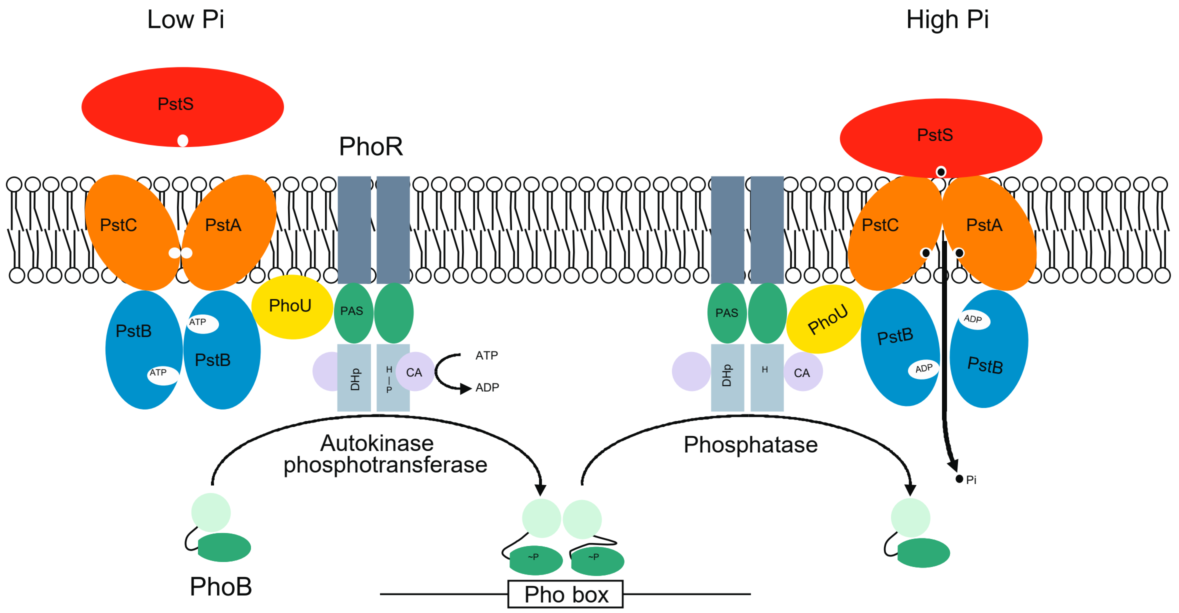

Supplementary Figure S1. Schematic representation of phosphate transport through the Pst transporter and signal transduction of PhoBR two component system in *E. coli*. When environmental phosphate is abundant, the Pst transporter signals through PhoU to induce PhoR phosphatase conformation. When Pi concentration is low, the transporter and PhoU induce PhoR autokinase conformation. The output of the signal transduction system relies on the phosphorylation state of the response regulator PhoB. Phospho-PhoB forms a dimer that binds specifically to DNA Pho boxes. Based on the model proposed by Gardner and McCleary (doi:10.1128/ecosalplus.ESP-0006-2019).

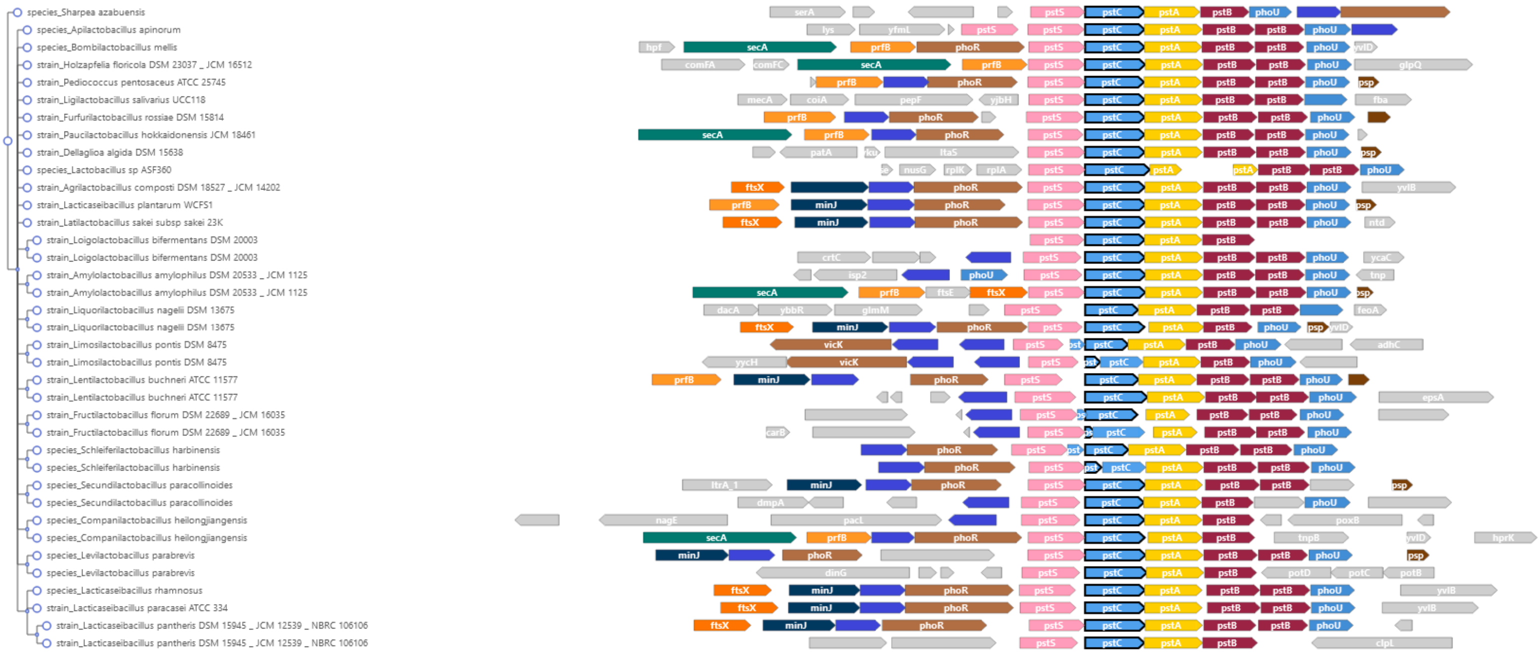

Supplementary Figure S2. Genomic context of *pstSCAB* genes in members of family *Lactobacillaceae*. The *phoP* genes, which in most cases precede the gene encoding the sensor kinase PhoR are depicted in dark blue color. In some strains, two *pstSCBA* operons exists, in which one of them is clustered with *phoPR* genes. The gene encoding a putative transmembrane protein with a PDZ domain, located upstream *phoP,* is annotated in most genomes as *minJ*, due to the homologies with MinJ from *Bacillus subtilis*. The comparisons were carried out with The Genomic Context Visualizer (GeCoViz; https://gecoviz.cgmlab.org/; Jorge Botas, Álvaro Rodríguez del Río, Joaquín Giner-Lamia, and Jaime Huerta-Cepas. (2022). GeCoViz: genomic context visualisation of prokaryotic genes from a functional and evolutionary perspective. Nucleic Acids Research, 50, W352–W357; doi.org/10.1093/nar/gkac367).

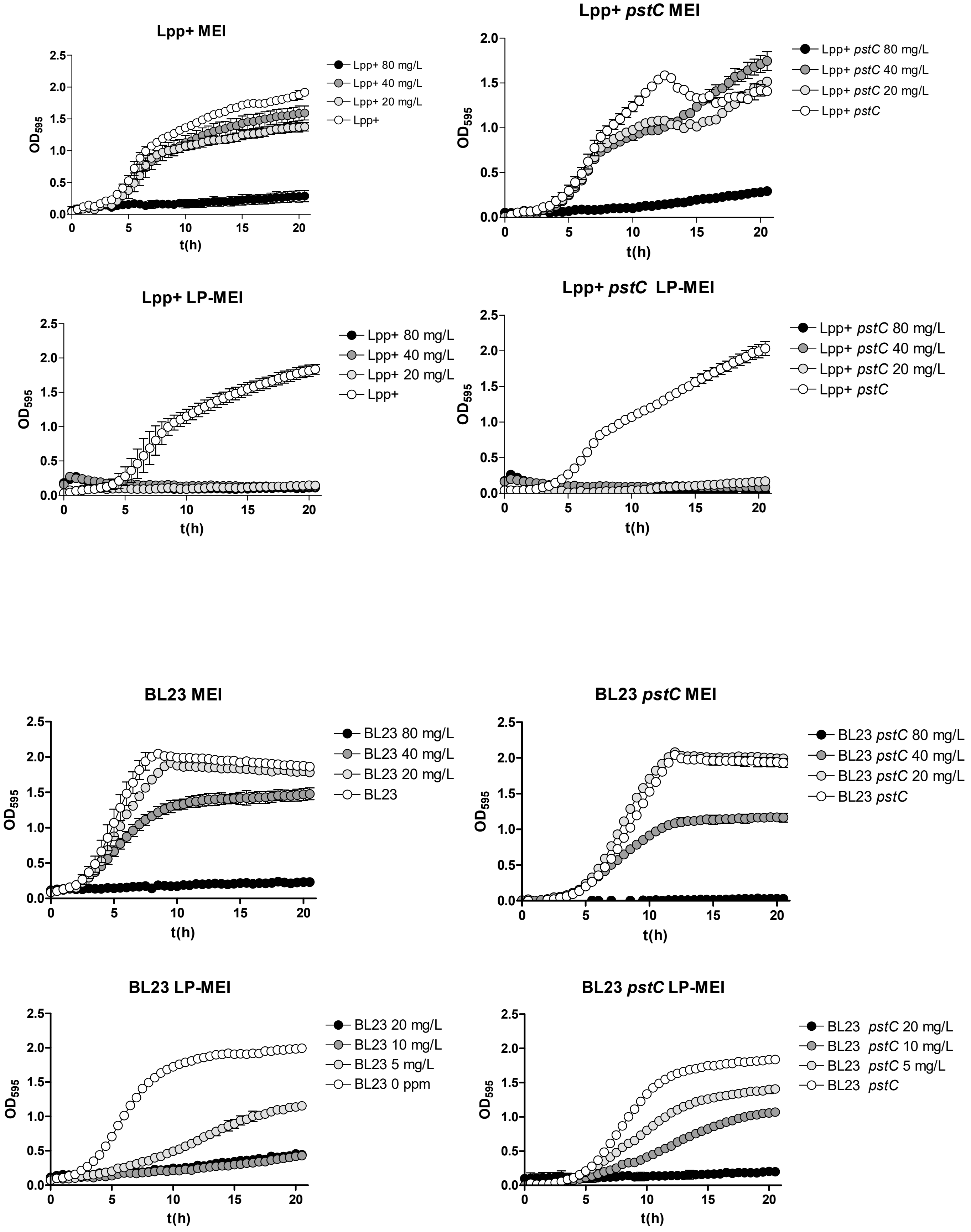

Supplementary Figure S3. Growth of *Lp. plantarum* Lpp+ and *Lc. paracasei* BL23 strains and their derivative *pstC* mutants in MEI and LP-MEI media with different concentrations of As(V).

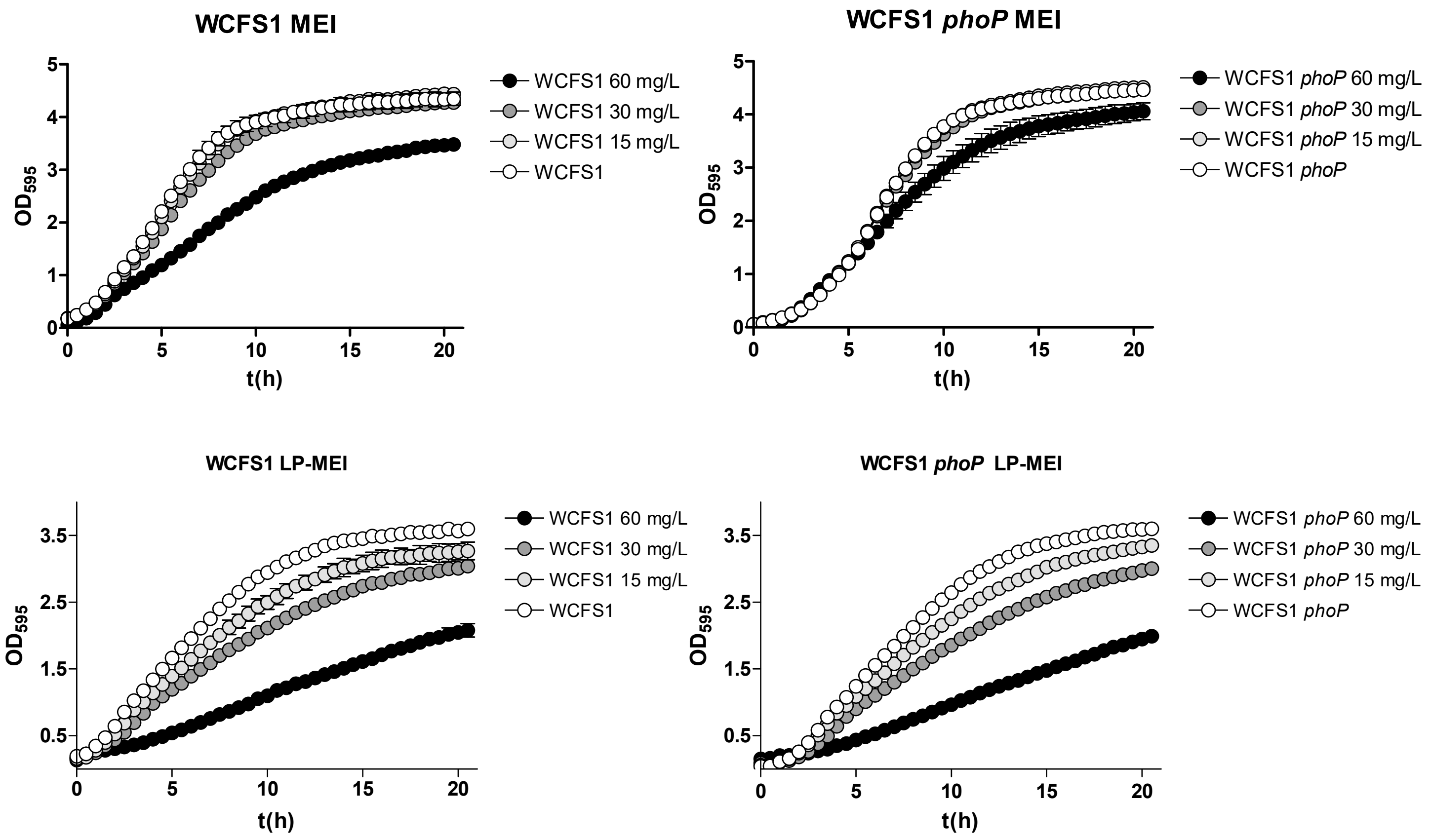

Supplementary Figure S4. Growth curves of *Lp. plantarum* WCFS1 and its derivative *phoP* mutant in MEI medium with different amounts of As(III).
