# Supplementary material for: Phosphate Uptake and Its Relation to Arsenic Toxicity in Lactobacilli
Source: Int J Mol Sci. 2024 May 4;25(9):5017. doi: 10.3390/ijms25095017 (PMC11084836; doi:10.3390/ijms25095017)
Supplement: Supplementary file 1 [file ijms-25-05017-s001.zip › Supplementary Table S1.pdf]

Supplementary Table S1. Oligonucleotides used in this study

Oligonucleotide	Sequence	Use
Lp. plantarum		
phoP-For	TTTTCTCGAGTGTGACGGCTAAAAATGATG	Disruption of *phoP* in *Lp. plantarum*
phoP-Rev	TTTTCTGCAGAAATTCCTTTGGTGTCAAGC	
phoP-C	CCACAAATTCGTAGCCAAAG	
pstC-For	TTTTCTCGAGTTCTACTTTGTTGCATCACG	Disruption of *pstC* in *Lp. plantarum*
pstC-Rev	TTTTCTGCAGAAAGGTCCCAGAAAGAATCC	
pstC-C	TTTATAAATTGTTTGCCAGCG	
phoU-For	TTTTCTCGAGACGGGAAGTTGAACTAGAAC	Disruption of *phoU* in *Lp. plantarum*
phoU-Rev	TTTTCTGCAGATACATCTCCCCGACTTTTG	
phoU-C	ATTCAGGTAATCCGTGGAAC	
Lc. paracasei		
pstCLcas-For	TCAAAGCTTCGCACGGCAATCCCGAAGTTG	Disruption of *pstC* in *Lc. paracasei* BL23
pstCLcas-Rev	TCAGAATTCCCGGAATCGCAGCACGTAGAACC	
pstCLcas-C	TCAGTTTAATTGTGATTGTGG	
phoULcas-For	ACGAAGCTTCGAATTGATTGCACTGCAG	Disruption of *phoU* in *Lc. paracasei* BL23
phoULcas-Rev	ATGGAATTCCATTGATCCCATGGTCTTC	
phoULcas-C	ATGAACGTGAAGTAGATTTGG	
dphoP-F1	ATATCGAATTCCTGCAGCCCAGGTGCGACCTTCATGACTC	Amplification of *phoP* 5' arm
dphoP-R1	TCGCGTAAATGGCTGACCTGCGCAGGTTCGTCATCAACAA	
dphoP-F2	TTGTTGATGACGAACCTGCGCAGGTCAGCCATTTACGCGA	Amplification of *phoP* 3' arm
dphoP-R2	CTAGAACTAGTGGATCCCCCGCAAAGGCAAGCGTTAGGAG	
dphoR-F1	ATATCGAATTCCTGCAGCCCCATAATCTAGATCGCATCCGTCG	Amplification of *phoR* 5' arm

dphoR-R1	CACAGGCAAAGTGACCGTGACATTCCCGACAAAGTCCAG	
dphoR-F2<br><br>dphoR-R2	CTGGACTTTGTCGGGAATGTCACGGTCACTTTGCCTGTG<br><br>CTAGAACTAGT<u>GGATCC</u>CCCCAGCTGTGCCATCGAGAC	Amplification of *phoR* 3' arm
CphoP-F<br><br>CphoP-R	atgttataatacaagtatcaTTTCCGGAGGAGACAGCATG<br><br>tttgttagcagccggatctaTTATTCATGGGGTGCCTCC	Cloning of *phoP* into pT1NX
CphoR-F<br><br>CphoR-R	atgttataatacaagtatcaATACCAAATGGAGGCACCC<br><br>tttgttagcagccggatctaTTTTTCATCCTCTGTGCGG	Cloning of *phoR* into pT1NX

The underlined nucleotides correspond to restriction sites introduced for cloning into pRV300. Oligonucleotides with a "-C" suffix in their names served to confirm plasmid integration at the correct locus. Nucleotides in lowercase are 5′ extensions for creating homology with pT1NX for the Gibson assembly reactions.
